# Supplementary material for: Unveiling the Role of Histone Methyltransferases in Psoriasis Pathogenesis: Insights from Transcriptomic Analysis
Source: Int J Mol Sci. 2025 Jun 30;26(13):6329. doi: 10.3390/ijms26136329 (PMC12249604; doi:10.3390/ijms26136329)
Supplement: Supplementary file 1 [file ijms-26-06329-s001.zip › ijms-3632577-Supplementary Information.pdf]

Supplementary Information

1.1 SET-domain methyltransferases with no or uncertain histone methyltransferase activity

In the case of SETD3, SETD4, SETD5 and SETD6, there is either a lack or uncertainty regarding their histone methyltransferase activity. In our analysis, we detected transcriptional differences of SETD3, SETD4, and SETD6 in the lesional skin, and for SETD5, in both non-lesional and lesional skin compared to healthy controls (Figure 1. d, Figure 2. and Supplementary Table 1.).

SETD3 was previously believed to methylate the H3K4 and K36 residues [1], but recent studies indicate that its sole substrate is only actin [2].

For SETD4, the potential ability to methylate various histone-based substrates is primarily based on either observation of methylation levels [3], which vary with changes in SETD4 expression, or *in vitro* methylation assays. The substrate specificity of SETD4 is influenced by the cellular environment and conditions that may or may not influence histone methylation [4].

In the case of SETD5, methyltransferase activity is absent, but instead, it organizes a corepressor complex that couples selective deacetylation of H3K9ac with methylation of this residue [5].

SETD6 exhibits structural and functional similarities to SETD3 [6], but may not possess histone methyltransferase activity, although it has been suggested to serve as a H2AZ monomethylates, methylating H2AZK7 during differentiation [7]. Therefore, altered expression of SETD6 may contribute to disturbances in self-renewal and differentiation [7] in psoriasis. However, non-histone methylation mediated by SETD6 regulates inflammatory responses in primary immune cells, governed by the RelA/NF-kappaB pathway [8,9]. Phosphorylated, active RelA/NF-kappaB levels are observed constitutively in non-lesional skin and are elevated in psoriatic basal epidermis, while active nuclear NF-kappaB is undetectable in healthy epidermis. Etanercept, a known therapeutic agent for psoriasis [10], reduces the activated RelA/NF-kappaB level, which correlates with reduced epidermal thickness and restoration of normal keratinocyte differentiation markers [11].

1.2. Seven-β-strand (7BS) methyltransferases

1.2.1 Seven-β-strand methyltransferases with histone lysine methylase activity

Among the seven-β-strand methyltransferases, only DOT1L and N6AMT1 (KMT9) possess histone lysine methyltransferase function [12,13] (Table 3). Among these two members, DOT1L showed lesion-associated changes in transcript levels in our analysis (Figure 1. d, Table 3. and Supplementary Table 1). It methylates all forms of histone H3 lysine 79 including, mono-, di- and trimethylation [13,14] (Table 3). DOT1L is a known regulator of cell proliferation, and plays a pivotal role in the G1/S phase transition and replication timing [15], and in the control of mitotic spindle formation and chromosome segregation [16]. Its activity preserves cell identity by collaborating with reprogramming-associated factors [17]. DOT1L orchestrates the commitment and differentiation of plasmacytoid dendritic cells [18] and Th2 cell lineage [19]. DOT1L also serves as a central player in preserving CD8+ T cells by averting premature differentiation and regulating epigenetic integrity [20]. Furthermore, DOT1L controls macrophage activation and the gene program for lipid biosynthesis [21]. Therefore, altered expression of DOT1L may play a critical role in the pathomechanism of psoriasis by modulating proliferation and immune responses.

1.2.2. Seven-β-strand lysine methyltransferases with non-histone modifying activity

Based on our current knowledge, some members of the seven-β-strand lysine methyltransferases do not possess histone-modifying activity [22,23]. These methyltransferases modify proteins of the cellular protein synthesis machinery, mitochondrial proteins, and molecular chaperones (Table 3.). In our study, we identified abnormal transcriptional expression in psoriatic skin from the seven-β-strand lysine methyltransferases EEF2KMT, METTL12, METTL13, METTL21A, and VCPKMT (Figure 1. d, Table 3. and Supplementary Table 1).

1.2.3. Seven-β-strand arginine methyltransferases: PRMT family

Members of the Protein Arginine Methyltransferase (PRMT) family [24] (Figure 1. b), exhibit dual functionality: they serve both as histone and non-histone methyltransferases [25,26]. PRMTs are classified into three primary types based on the specific form of methylated arginine they produce (PRMTI-III) [27]. The Type I PRMTs predominantly target histones H3 and H4 for methylation, whereas PRMT5, classified as a Type II PRMT, methylates nearly all histone forms

with the exception of H2B. While Type III PRMTs possess the ability to methylate all histone forms (Figure 3 and Table 4).

PRMT-mediated arginine methylation of histones and non-histone proteins [25-27] can influence numerous cellular processes, including the cell cycle [28], translation, splicing [29], and signal transduction [30]. In addition, in primary T cells, PRMT-mediated methylation of proteins is crucial for cell fate determination and differentiation [31]. Therefore, their altered functioning may influence proliferation and differentiation abnormalities related to psoriasis, including keratinocyte proliferation and the T cell-mediated immune responses.

Type I PRMTs, primarily catalyze asymmetric arginine dimethylation of proteins [27]. Among these, our analysis revealed differential expression of CARM1, PRMT1, and PRMT2 in lesional skin (Figure 1 d, Figure 3., Table 4 and Supplementary Table 1).

CARM1 is known to mediate glucocorticoid-induced inhibition of keratin K6/K16 transcription and β-catenin-mediated keratinocyte migration in chronic wound healing [32,33]. CARM1 plays a crucial role in IFN-γ-mediated MHC II gene expression [34] and thymopoiesis [35].

PRMT1 plays a critical role in maintaining epidermal progenitor cells by regulating their self-renewal and differentiation processes [36]. It may also affect skin structure by modulating cellular processes involved in cytoskeletal dynamics and cellular adhesion [37]. The methylation activity of PRMT1 regulates cytokine production in T cells [38], including the production of IL-17 cytokine by Th17 cells [39]. PRMT1 negatively regulate MHC II macrophage antigen presentation [40] and TNFα-triggered NF-κB activation [41]. PRMT1 and CARM1 have opposing roles in the regulation of CXCL10 expression, with PRMT1 acting as a negative [41], and CARM1 as a positive regulator [28,42], CXCL10 is implicated in the pathogenesis of psoriasis, and its levels in serum are often associated with disease activity, particularly in the context of Th1-mediated inflammation [43].

PRMT2 regulates the cell cycle by modulating E2F activity [44], while it controls interferon-β production through TLR4/IRF3 pathways [45]. This modulatory function of PRMT2 may be important in the development of psoriasis. In addition, resveratrol, a promising anti-psoriatic agent [46], may alleviate lipopolysaccharide-induced inflammation by blocking the activation of the TLR4-NF-κB/MAPKs/IRF3 signaling cascade governed by PRMT2 [47].

Type II PRMTs are responsible for mediating symmetric arginine demethylation [27], among which we detected abnormal expression of PRMT5 in both non-lesional and lesional skin (Figure 1. d; Figure 3., Table 4. and Supplementary Table 1.).

The formation of the PRMT5-MEP50 complex in response to antiproliferative signals contributes to the inhibition of proliferation during keratinocyte differentiation [48]. In the context of the innate immune system, PRMT5 facilitates MHC II transcription in macrophages in an enzyme activity-dependent manner [49]. In addition, given its importance in epidermal development and its multifaceted role in T cell biology, that includes thymic T cell production, activation-induced proliferation, peripheral T cell homeostasis, and Th17 differentiation [50]. PRMT5 may play a significant function in psoriasis.

Type III PRMTs exclusively catalyze monomethyl-arginine formation, with PRMT7 being the only member [27]. While PRMT7 is expressed normally in non-lesional skin, its expression is affected in lesions (Figure 1. d, Figure 3., Table 4 and Supplementary Table 1). PRMT7 directly regulates pluripotent transcription factors [51], while also inhibiting cellular senescence [52,53]. PRMT7 modulate adhesion and migratory capacity of monocytes [54,55], thereby may contribute to the heightened adhesion and aggregation of monocytes in psoriatic skin [56].

Supplementary Reference

1. Eom GH, Kim K-B, Kim JH, Kim J-Y, Kim J-R, Kee HJ, et al. Histone methyltransferase SETD3 regulates muscle differentiation. J Biol Chem 2011;286:34733–42. https://doi.org/10.1074/jbc.M110.203307.
2. Kwiatkowski S, Seliga AK, Vertommen D, Terreri M, Ishikawa T, Grabowska I, et al. SETD3 protein is the actin-specific histidine N-methyltransferase. eLife 2018;7. https://doi.org/10.7554/eLife.37921.
3. Wu C, Wu L, Ha Y, Zou Y, Shi K, Xing J, et al. Methyltransferase SETD4 mediates macrophages proliferation through EGFR signaling 2023. https://doi.org/10.21203/rs.3.rs-1958184/v3.
4. Wang Y, Shen Z. Unmasking the mammalian SET domain-containing protein 4. NAR Cancer 2022;4:zcac021. https://doi.org/10.1093/narcan/zcac021.
5. Li M, Hou Y, Zhang Z, Zhang B, Huang T, Sun A, et al. Structure, activity and function of the lysine methyltransferase SETD5. Front Endocrinol (Lausanne) 2023;14:1089527. https://doi.org/10.3389/fendo.2023.1089527.
6. Admoni-Elisha L, Abaev-Schneiderman E, Cohn O, Shapira G, Shomron N, Feldman M, et al. Structure-function conservation between the methyltransferases SETD3 and SETD6. Biochimie 2022;200:27–35. https://doi.org/10.1016/j.biochi.2022.05.003.
7. Binda O, Sevilla A, LeRoy G, Lemischka IR, Garcia BA, Richard S. SETD6 monomethylates H2AZ on lysine 7 and is required for the maintenance of embryonic stem cell self-renewal. Epigenetics 2013;8:177–83. https://doi.org/10.4161/epi.23416.
8. Chang Y, Levy D, Horton JR, Peng J, Zhang X, Gozani O, et al. Structural basis of SETD6-mediated regulation of the NF-kB network via methyl-lysine signaling. Nucleic Acids Res 2011;39:6380–9. https://doi.org/10.1093/nar/gkr256.
9. Levy D, Kuo AJ, Chang Y, Schaefer U, Kitson C, Cheung P, et al. SETD6 lysine methylation of RelA couples GLP activity at chromatin to tonic repression of NF-κB signaling. Nat Immunol 2011;12:29–36. https://doi.org/10.1038/ni.1968.
10. Nguyen TU, Koo J. Etanercept in the treatment of plaque psoriasis. Clin Cosmet Investig Dermatol 2009;2:77–84.
11. Lizzul PF, Aphale A, Malaviya R, Sun Y, Masud S, Dombrovskiy V, et al. Differential expression of phosphorylated NF-kappaB/RelA in normal and psoriatic epidermis and downregulation of NF-kappaB in response to treatment with etanercept. J Invest Dermatol 2005;124:1275–83. https://doi.org/10.1111/j.0022-202X.2005.23735.x.
12. Metzger E, Wang S, Urban S, Willmann D, Schmidt A, Offermann A, et al. KMT9 monomethylates histone H4 lysine 12 and controls proliferation of prostate cancer cells. Nat Struct Mol Biol 2019;26:361–71. https://doi.org/10.1038/s41594-019-0219-9.
13. Min J, Feng Q, Li Z, Zhang Y, Xu R-M. Structure of the Catalytic Domain of Human DOT1L, a Non-SET Domain Nucleosomal Histone Methyltransferase. Cell 2003;112:711–23. https://doi.org/10.1016/S0092-8674(03)00114-4.
14. van Leeuwen F, Gafken PR, Gottschling DE. Dot1p Modulates Silencing in Yeast by Methylation of the Nucleosome Core. Cell 2002;109:745–56. https://doi.org/10.1016/S0092-8674(02)00759-6.
15. Kim W, Choi M, Kim J-E. The histone methyltransferase Dot1/DOT1L as a critical regulator of the cell cycle. Cell Cycle 2014;13:726–38. https://doi.org/10.4161/cc.28104.

16. Kim W, Kim R, Park G, Park J-W, Kim J-E. Deficiency of H3K79 histone methyltransferase Dot1-like protein (DOT1L) inhibits cell proliferation. J Biol Chem 2012;287:5588–99. https://doi.org/10.1074/jbc.M111.328138.
17. Wille CK, Sridharan R. DOT1L inhibition enhances pluripotency beyond acquisition of epithelial identity and without immediate suppression of the somatic transcriptome. Stem Cell Reports 2022;17:384–96. https://doi.org/10.1016/j.stemcr.2021.12.004.
18. Tian Y, Meng L, Yu H, Hexner EO, Zheng L, Hu S, et al. Graft-Versus-Host Disease Impairs the Histone Methyltransferase Dot1l-Regulated Reconstitution of Plasmacytoid Dendritic Cells in Mice Undergoing Allo-HSCT. Blood 2018;132:477. https://doi.org/10.1182/blood-2018-99-118751.
19. Scheer S, Runting J, Bramhall M, Russ B, Zaini A, Ellemor J, et al. The Methyltransferase DOT1L Controls Activation and Lineage Integrity in CD4+ T Cells during Infection and Inflammation. Cell Rep 2020;33:108505. https://doi.org/10.1016/j.celrep.2020.108505.
20. Kwesi-Maliepaard EM, Aslam MA, Alemdehy MF, van den Brand T, McLean C, Vlaming H, et al. The histone methyltransferase DOT1L prevents antigen-independent differentiation and safeguards epigenetic identity of CD8+ T cells. Proceedings of the National Academy of Sciences 2020;117:20706–16. https://doi.org/10.1073/pnas.1920372117.
21. Willemsen L, Prange KHM, Neele AE, van Roomen CPAA, Gijbels M, Griffith GR, et al. DOT1L regulates lipid biosynthesis and inflammatory responses in macrophages and promotes atherosclerotic plaque stability. Cell Rep 2022;41:111703. https://doi.org/10.1016/j.celrep.2022.111703.
22. Falnes PØ, Małecki JM, Herrera MC, Bengtsen M, Davydova E. Human seven-β-strand (METTL) methyltransferases - conquering the universe of protein lysine methylation. J Biol Chem 2023;299:104661. https://doi.org/10.1016/j.jbc.2023.104661.
23. Lukinović V, Casanova AG, Roth GS, Chuffart F, Reynoird N. Lysine Methyltransferases Signaling: Histones are Just the Tip of the Iceberg. Curr Protein Pept Sci 2020;21:655–74. https://doi.org/10.2174/1871527319666200102101608.
24. Yang Y, Bedford MT. Protein arginine methyltransferases and cancer. Nat Rev Cancer 2013;13:37–50. https://doi.org/10.1038/nrc3409.
25. Al-Hamashi AA, Diaz K, Huang R. Non-Histone Arginine Methylation by Protein Arginine Methyltransferases. Curr Protein Pept Sci 2020;21:699–712. https://doi.org/10.2174/1389203721666200507091952.
26. Zheng K, Chen S, Ren Z, Wang Y. Protein arginine methylation in viral infection and antiviral immunity. International Journal of Biological Sciences 2023;19:5292–318. https://doi.org/10.7150/ijbs.89498.
27. Blanc RS, Richard S. Arginine Methylation: The Coming of Age. Molecular Cell 2017;65:8–24. https://doi.org/10.1016/j.molcel.2016.11.003.
28. Raposo AE, Piller SC. Protein arginine methylation: an emerging regulator of the cell cycle. Cell Div 2018;13:3. https://doi.org/10.1186/s13008-018-0036-2.
29. Wei H-H, Fan X-J, Hu Y, Tian X-X, Guo M, Mao M-W, et al. A systematic survey of PRMT interactomes reveals the key roles of arginine methylation in the global control of RNA splicing and translation. Science Bulletin 2021;66:1342–57. https://doi.org/10.1016/j.scib.2021.01.004.
30. Bedford MT, Richard S. Arginine methylation an emerging regulator of protein function. Mol Cell 2005;18:263–72. https://doi.org/10.1016/j.molcel.2005.04.003.

31. Geoghegan V, Guo A, Trudgian D, Thomas B, Acuto O. Comprehensive identification of arginine methylation in primary T cells reveals regulatory roles in cell signalling. Nat Commun 2015;6:6758. https://doi.org/10.1038/ncomms7758.
32. Stojadinovic O, Brem H, Vouthounis C, Lee B, Fallon J, Stallcup M, et al. Molecular Pathogenesis of Chronic Wounds: The Role of β-Catenin and c-myc in the Inhibition of Epithelialization and Wound Healing. The American Journal of Pathology 2005;167:59–69. https://doi.org/10.1016/S0002-9440(10)62953-7.
33. Stojadinovic O, Vouthounis C, Lee B, Stallcup M, Tomic-Canic M. 142 β-Catenin and Carm-1 as Co-Repressors of Glucocorticoid Receptor Lead to Inhibition of Keratinocyte Migration. Wound Repair and Regeneration 2004;12:A37–A37. https://doi.org/10.1111/j.1067-1927.2004.0abstractek.x.
34. Zika E, Fauquier L, Vandel L, Ting JP-Y. Interplay among coactivator-associated arginine methyltransferase 1, CBP, and CIITA in IFN-gamma-inducible MHC-II gene expression. Proc Natl Acad Sci U S A 2005;102:16321–6. https://doi.org/10.1073/pnas.0505045102.
35. Li J, Zhao Z, Carter C, Ehrlich LIR, Bedford MT, Richie ER. CARM1 regulates fetal hematopoiesis and thymocyte development. J Immunol 2013;190:597–604. https://doi.org/10.4049/jimmunol.1102513.
36. Bao X, Siprashvili Z, Zarnegar BJ, Shenoy RM, Rios EJ, Nady N, et al. CSNK1a1 Regulates PRMT1 to Maintain the Progenitor State in Self-Renewing Somatic Tissue. Dev Cell 2017;43:227-239.e5. https://doi.org/10.1016/j.devcel.2017.08.021.
37. Albrecht LV, Zhang L, Shabanowitz J, Purevjav E, Towbin JA, Hunt DF, et al. GSK3- and PRMT-1–dependent modifications of desmoplakin control desmoplakin–cytoskeleton dynamics. Journal of Cell Biology 2015;208:597–612. https://doi.org/10.1083/jcb.201406020.
38. Mowen KA, Schurter BT, Fathman JW, David M, Glimcher LH. Arginine Methylation of NIP45 Modulates Cytokine Gene Expression in Effector T Lymphocytes. Molecular Cell 2004;15:559–71. https://doi.org/10.1016/j.molcel.2004.06.042.
39. Sen S, He Z, Ghosh S, Dery KJ, Yang L, Zhang J, et al. PRMT1 Plays a Critical Role in Th17 Differentiation by Regulating Reciprocal Recruitment of STAT3 and STAT5. The Journal of Immunology 2018;201:440–50. https://doi.org/10.4049/jimmunol.1701654.
40. Fan Z, Li J, Li P, Ye Q, Xu H, Wu X, et al. Protein arginine methyltransferase 1 (PRMT1) represses MHC II transcription in macrophages by methylating CIITA. Sci Rep 2017;7:40531. https://doi.org/10.1038/srep40531.
41. Reintjes A, Fuchs JE, Kremser L, Lindner HH, Liedl KR, Huber LA, et al. Asymmetric arginine dimethylation of RelA provides a repressive mark to modulate TNFα/NF-κB response. Proc Natl Acad Sci U S A 2016;113:4326–31. https://doi.org/10.1073/pnas.1522372113.
42. Covic M, Hassa P, Saccani S, Buerki C, Meier N, Lombardi C, et al. Arginine methyltransferase CARM1 is a promoter-specific regulator of NF-κB-dependent gene expression. The EMBO Journal 2005;24:85–96. https://doi.org/10.1038/sj.emboj.7600500.
43. Ferrari SM, Ruffilli I, Colaci M, Antonelli A, Ferri C, Fallahi P. CXCL10 in psoriasis. Advances in Medical Sciences 2015;60:349–54. https://doi.org/10.1016/j.advms.2015.07.011.
44. Yoshimoto T, Boehm M, Olive M, Crook MF, San H, Langenickel T, et al. The arginine methyltransferase PRMT2 binds RB and regulates E2F function. Experimental Cell Research 2006;312:2040–53. https://doi.org/10.1016/j.yexcr.2006.03.001.

45. Wang J, Hua H, Wang F, Yang S, Zhou Q, Wu X, et al. Arginine methylation by PRMT2 promotes IFN-β production through TLR4/IRF3 signaling pathway. Molecular Immunology 2021;139:202–10. https://doi.org/10.1016/j.molimm.2021.08.014.
46. Oliveira AL de B, Monteiro VVS, Navegantes-Lima KC, Reis JF, Gomes R de S, Rodrigues DVS, et al. Resveratrol Role in Autoimmune Disease—A Mini-Review. Nutrients 2017;9:1306. https://doi.org/10.3390/nu9121306.
47. Tong W, Chen X, Song X, Chen Y, Jia R, Zou Y, et al. Resveratrol inhibits LPS-induced inflammation through suppressing the signaling cascades of TLR4-NF-κB/MAPKs/IRF3. Experimental and Therapeutic Medicine 2020;19:1824–34. https://doi.org/10.3892/etm.2019.8396.
48. Saha K, Eckert RL. Methylosome Protein 50 and PKCδ/p38δ Protein Signaling Control Keratinocyte Proliferation via Opposing Effects on p21Cip1 Gene Expression *. Journal of Biological Chemistry 2015;290:13521–30. https://doi.org/10.1074/jbc.M115.642868.
49. Fan Z, Kong X, Xia J, Wu X, Li H, Xu H, et al. The arginine methyltransferase PRMT5 regulates CIITA-dependent MHC II transcription. Biochimica et Biophysica Acta (BBA) - Gene Regulatory Mechanisms 2016;1859:687–96. https://doi.org/10.1016/j.bbagrm.2016.03.004.
50. Sengupta S, Kennemer A, Patrick K, Tsichlis P, Guerau-de-Arellano M. Protein Arginine Methyltransferase 5 in T Lymphocyte Biology. Trends in Immunology 2020;41:918–31. https://doi.org/10.1016/j.it.2020.08.007.
51. Lee S-H, Chen T-Y, Dhar SS, Gu B, Chen K, Kim YZ, et al. A feedback loop comprising PRMT7 and miR-24-2 interplays with Oct4, Nanog, Klf4 and c-Myc to regulate stemness. Nucleic Acids Research 2016;44:10603–18. https://doi.org/10.1093/nar/gkw788.
52. Vuong TA, Jeong H-J, Lee H-J, Kim B-G, Leem Y-E, Cho H, et al. PRMT7 methylates and suppresses GLI2 binding to SUFU thereby promoting its activation. Cell Death Differ 2020;27:15–28. https://doi.org/10.1038/s41418-019-0334-5.
53. Blanc RS, Vogel G, Chen T, Crist C, Richard S. PRMT7 Preserves Satellite Cell Regenerative Capacity. Cell Rep 2016;14:1528–39. https://doi.org/10.1016/j.celrep.2016.01.022.
54. Jeridi A, Conlon TM, Günsel GG, Lang NJ, Burgstaller G, Eeckhoutte HPV, et al. Monocyte migration and COPD pathogenesis are epigenetically regulated by PRMT7. ERJ Open Research 2022;8. https://doi.org/10.1183/23120541.LSC-2022.178.
55. Günes Günsel G, Conlon TM, Jeridi A, Kim R, Ertüz Z, Lang NJ, et al. The arginine methyltransferase PRMT7 promotes extravasation of monocytes resulting in tissue injury in COPD. Nat Commun 2022;13:1303. https://doi.org/10.1038/s41467-022-28809-4.
56. Golden JB, Groft SG, Squeri MV, Debanne SM, Ward NL, McCormick TS, et al. Chronic psoriatic skin inflammation leads to increased monocyte adhesion and aggregation. J Immunol 2015;195:2006–18. https://doi.org/10.4049/jimmunol.1402307.
